# Effect of betaine supplementation on plasma nitrate/nitrite in exercise-trained men

**DOI:** 10.1186/1550-2783-8-5

**Published:** 2011-03-18

**Authors:** Richard J Bloomer, Tyler M Farney, John F Trepanowski, Cameron G McCarthy, Robert E Canale

**Affiliations:** 1 Cardiorespiratory/Metabolic Laboratory, The University of Memphis Memphis, TN 38152, USA

## Abstract

**Background:**

Betaine, beetroot juice, and supplemental nitrate have recently been reported to improve certain aspects of exercise performance, which may be mechanistically linked to increased nitric oxide. The purpose of the present study was to investigate the effect of betaine supplementation on plasma nitrate/nitrite, a surrogate marker or nitric oxide, in exercise-trained men.

**Methods:**

We used three different study designs (acute intake of betaine at 1.25 and 5.00 grams, chronic intake of betaine at 2.5 grams per day for 14 days, and chronic [6 grams of betaine per day for 7 days] followed by acute intake [6 grams]), all involving exercise-trained men, to investigate the effects of orally ingested betaine on plasma nitrate/nitrite. Blood samples were collected before and at 30, 60, 90, and 120 min after ingestion of 1.25 and 5.00 grams of betaine (Study 1); before and after 14 days of betaine supplementation at a dosage of 2.5 grams (Study 2); and before and after 7 days of betaine supplementation at a dosage of 6 grams, followed by acute ingestion of 6 grams and blood measures at 30 and 60 min post ingestion (Study 3).

**Results:**

In Study 1, nitrate/nitrite was relatively unaffected and no statistically significant interaction (p = 0.99), dosage (p = 0.69), or time (p = 0.91) effects were noted. Similar findings were noted in Study 2, with no statistically significant interaction (p = 0.57), condition (p = 0.98), or pre/post intervention (p = 0.17) effects noted for nitrate/nitrite. In Study 3, no statistically significant changes were noted in nitrate/nitrite between collection times (p = 0.97).

**Conclusion:**

Our data indicate that acute or chronic ingestion of betaine by healthy, exercise-trained men does not impact plasma nitrate/nitrite. These findings suggest that other mechanisms aside from increasing circulating nitric oxide are likely responsible for any performance enhancing effect of betaine supplementation.

## Background

Betaine (chemically known as 2-(Trimethylammonio) ethanoic acid, hydroxide, inner salt) is isolated from sugar beets and sold for a variety of uses, including animal feed, as a food ingredient, and as a dietary supplement. Betaine has several noted effects related to human health and function, including acting as an osmolyte (protecting cells against dehydration [1]), as an antioxidant agent (protecting cells against free radicals) [2], as a methyl group donor (lowering potentially harmful levels of homocysteine [3]), and as a vascular protectant [4].

Although traditionally not used for purposes of exercise performance, over the past few years investigators have reported positive findings for betaine in this regard. For example, the powdered form of betaine has been noted to improve certain aspects of exercise performance when active college-aged men ingested a dosage of 2.5 grams per day for 14 [5] or 15 days [6]. We have recently completed a study which corroborates these findings (unpublished data). Moreover, recent studies using either beetroot juice (500 mL/day--providing approximately 340 mg of dietary nitrate) [7-9] or sodium nitrate [10] have noted favorable outcomes pertaining to endurance exercise performance, while also noting a significant increase in plasma nitrite levels [7-9].

Although the mechanism for the ergogenic effect of betaine itself has yet to be elucidated, it has been suggested that improvements in exercise performance following nitrate ingestion may be at least partially associated with the increase in the production/availability of nitric oxide [7,8]. More recently, it has been noted that nitrate supplementation improves mitochondrial efficiency in healthy human subjects [11], which may provide additional mechanistic data pertaining to an ergogenic effect. Nitric oxide, which is synthesized in the body from L-arginine, oxygen, and a variety of other cofactors by a family of enzymes known as nitric oxide synthases [12], was originally referred to as endothelium-derived relaxing factor [13], due to its ability to promote vasorelaxion of smooth muscle.

While nitric oxide has numerous other functions within the human body [14,15], in relation to sport nutrition and "nitric oxide stimulating dietary supplements", the *potential *for nitric oxide to promote an increase in blood flow to the working muscles appears of most interest. While direct evidence is not available to support this notion, the widespread theory within the sport nutrition industry is that an increase in circulating nitric oxide would lead to an increase in blood flow to exercising muscle, which in turn would lead to an increase in nutrient delivery *to *and waste removal *from *that muscle, leading to enhanced exercise performance and recovery. Of course, this is largely speculation, as has been discussed in more detail recently [16].

With regards to betaine and the potential for increasing nitric oxide, a study by Iqbal and colleagues found that daily supplementation at an oral dosage of 6 grams for 7 days, followed by a single serving on day 8 of 3 grams, had a profound effect (20-90%) on elevating blood nitrate/nitrite, a surrogate marker of nitric oxide [17]. Similar results were reported by Iqbal and coworkers in another study [18]. However, aside from these studies (available only as abstracts and within a US patent application [US 2007/2013399 A1], and not in manuscript form), no published investigations have focused on the effect of betaine to elevate nitrate/nitrite. Therefore, the purpose of our work was to investigate the effects of orally ingested betaine in exercise-trained men (the most likely candidates for use of betaine as an ergogenic aid) using three different study designs (acute intake at two different dosages, chronic intake at one dosage, and chronic followed by acute intake--as to replicate the work of Iqbal et al.). We hypothesized that betaine ingestion would increase plasma nitrate/nitrite levels, in a manner consistent with the findings of Iqbal and coworkers [17,18].

## Methods

### Subjects

Subjects for all three studies were recruited from the University of Memphis Campus and surrounding community. Subjects were allowed to participate in more than one study. However, this was only the case for a few of the subjects. Study 1 was completed first, followed by an approximate one month break before beginning Study 2. Study 3 was started approximately five months after the completion of Study 2. Subjects were not smokers, did not have self-reported cardiovascular or metabolic disease, and were exercise-trained. Subjects were not using dietary supplements believed to influence blood nitrate/nitrite. That is, subjects were allowed to continue their normal intake of multi-vitamin/mineral supplements, as well as protein powder. Characteristics of subjects are presented in Table 1. Health history, drug and dietary supplement usage, and physical activity questionnaires were completed by subjects to determine eligibility. Subjects were instructed to maintain their current exercise and dietary intake programs throughout the study periods. However, in all three studies subjects were instructed to refrain from strenuous exercise during the 24 hours prior to each test session, and to avoid intake of nitrate rich foods (e.g., cured meats, beets, spinach). All studies were approved by the university committee for human subject research (H10-43; H10-44; H11-09) and all subjects provided written consent.

**Table 1:** Characteristics of exercise-trained men

Variable	Study 1 N = 8	Study 2 N = 13	Study 3 N = 10
Age (yrs)	25 ± 6	23 ± 3	27 ± 5
Height (cm)	178 ± 6	178 ± 8	178 ± 3
Weight (kg)	86 ± 15	82 ± 12	81 ± 7
BMI (kg∙m^-2^)	27 ± 4	26 ± 4	26 ± 3
Body fat (%)	17 ± 5	15 ± 7	13 ± 6
Waist:Hip	0.81 ± 0.05	0.84 ± 0.04	0.82 ± 0.04
Resting heart rate (bpm)	66 ± 5	68 ± 5	62 ± 10
Resting SBP (mmHg)	117 ± 6	114 ± 11	111 ± 11
Resting DBP (mmHg)	74 ± 8	73 ± 9	61 ± 8
Years resistance exercise training	7 ± 8	4 ± 3	7 ± 6
Hours per week resistance exercise	4 ± 3	4 ± 1	4 ± 1
Years aerobic exercise training	4 ± 4	3 ± 3	5 ± 4
Hours per week aerobic exercise	2 ± 1	2 ± 2	2 ± 1

Data are mean ± SD.

Study 1: cross-over design with subjects consuming either 1.25 or 5.00 grams of betaine in a single ingestion.

Study 2: cross-over design with subjects consuming 2.5 grams of betaine or a placebo daily for 14 days; 21 day washout period between each condition.

Study 3: subjects consumed 6 grams of betaine daily for 7 days.

### Screening

For all studies, during the initial visit to the laboratory, subjects completed the informed consent form, health and physical activity questionnaires. Subjects' heart rate and blood pressure, height, weight, waist and hip circumference, and skinfold thickness (7 site) was measured and used for descriptive purposes. Subjects were provided with food logs and instructions regarding how to complete these logs during the day prior to each test day.

### Testing

For all studies, subjects reported to the laboratory in the morning hours (6:00-9:00 am) following a 10 hour overnight fast. Upon arrival to the lab, subjects rested for 10 minutes. The betaine used in all studies was delivered in powder form (BetaPower™; 99% pure betaine anhydrous; Danisco; Copenhagen, Denmark). Specific procedures for each of the three studies are provided below.

#### Study 1

Effect of acute ingestion of betaine at two different dosages on plasma nitrate/nitrite: Subjects reported to the lab on two different days separated by one week. During both visits subjects consumed betaine mixed in 240 mL of water at a dosage of either 1.25 or 5.00 grams. The order of the dosing was randomized and subjects were blind to the dosage. Blood samples were taken before (after the 10 minute quiet rest period) and at 30, 60, 90, and 120 minutes following ingestion in order to determine the effect of a single dosage of betaine on plasma nitrate/nitrite. No food or calorie containing beverages were allowed during the test period, although water was allowed *ad libitum *and matched for each subject during both days of testing.

#### Study 2

Effect of chronic ingestion of betaine on plasma nitrate/nitrite: Subjects were randomly assigned in double-blind manner using a cross-over design to betaine (2.5 grams of betaine powder mixed into 500 mL of Gatorade^®^) or placebo (500 mL of Gatorade^®^). Subjects were instructed to consume 250 mL twice per day. Betaine powder is tasteless to most individuals when mixed into 500 mL of Gatorade^®^. To better ensure that subjects consumed the entire dosage of 2.5 grams of betaine each day, without the need for subjects to mix the betaine into a beverage themselves, Gatorade^® ^was used as a delivery vehicle--as the betaine could be added to 500 mL Gatorade^® ^bottles by investigators and distributed to subjects for the entire 14 day period. This has been done in prior work with betaine [5,6]. The treatment period for both conditions was 14 days and a 21 day washout period was included between conditions. Blood samples were taken before and after each 14 day treatment period (after the 10 minute quiet rest period) in order to determine the effect of chronic supplementation with betaine on plasma nitrate/nitrite.

#### Study 3

Effect of chronic followed by acute ingestion of betaine on plasma nitrate/nitrite: Subjects reported to the laboratory on day 1 and day 8. On day 1, subjects simply provided a fasting, resting blood sample. They were then provided with individual servings of betaine (3 grams per serving) and instructed to ingest two servings per day (6 grams total) for seven days, mixed in water. Subjects returned to the lab on day 8 and a fasting, resting blood sample was obtained. Subjects then ingested 6 grams of betaine mixed into 150 mL of water. Rather than use Gatorade^®^, as was done in Study 2, we chose to use water only (at a lower volume), in an attempt to more closely mimic the work of Iqbal and coworkers [17]. Additional blood samples were taken at 30 and 60 minutes post ingestion. No food or calorie containing beverages were allowed during the test period, although water was allowed *ad libitum *and matched for each subject during both days of testing. This design allowed us to determine both the chronic and acute effects of betaine ingestion of plasma nitrate/nitrite. This third design differed from designs 1 and 2 in that we used a higher dosage of betaine during the chronic supplementation period, and while the 6 gram acute dosage was not much different than the 5 gram acute dosage provided in Study 1, this was preceded by a 7 day treatment period with 6 grams of betaine per day. In comparison, Study 1 simply used a single ingestion of betaine without any pretreatment period. It should be noted that while we attempted to mimic as closely as possible the design of Iqbal and colleagues [17], due to the fact that their work was not presented in peer reviewed manuscript format, it is possible that some design differences did occur between our study and their work.

### Blood Processing and Biochemistry

At each time of blood collection, venous samples (~7 mL) were taken from an antecubital vein via needle and Vacutainer^®^. Repeated venipunctures were used for blood collection in all studies. We have noted in prior work using resistance trained men as subjects that performing repeated venipunctures is not associated with problems in obtaining blood samples. Moreover, we have compared the use of repeated venipunctures with the use of indwelling catheter placement on serial blood sample collection over time, and have noted no difference in terms of endothelial cell derived peptides (e.g., endothelin-1 [19]).

Following collection, whole blood samples were placed immediately in a refrigerated centrifuge and processed (1500 *g *for 15 minutes at 4°C) in order to separate the plasma from cells. Plasma was then stored at -70°C until analyzed for nitrate/nitrite using a commercially available colorimetric assay kit (Catalog#: 780001; Caymen Chemical, Ann Arbor, MI), according to the procedures provided by the manufacturer. After being thawed, plasma samples were centrifuged at 10,000 *g *for 5 minutes in a refrigerated centrifuge (4°C). Following the addition of a nitrate reductase co-factor to each diluted sample, nitrate reductase was added and the mixture was incubated for three hours to allow for the full conversion of nitrate to nitrite. Greiss reagent was then added, which converts nitrite into a deep purple azo compound. The absorbance was then detected at 540 nm using a PowerWave microplate spectrophotometer (BioTek Instruments, Winooski, VT). Quantification was performed with a calibration curve. The coefficient of variation for this assay in our laboratory is <8%. The detection limit, as per the manufacturer, is ≥2.5 μM. It should be noted that the products of nitric oxide metabolism, nitrate (NO_3_^-^) and nitrite (NO_2_^-^), are typically measured in blood samples due to the short half life of nitric oxide (i.e., equal to only 3-4 seconds). For Study 3, in addition to total nitrate/nitrite, nitrite only was measured using the same procedures outlined above, with the exclusion of nitrate reductase co-factor and nitrate reductase. The measurement of nitrite was done as an afterthought following the analysis of nitrate/nitrite. Our rationale for including the sole measure of nitrite in Study 3 was based on recent findings for beetroot juice and nitrite elevation [7-9]. We believed that of all three studies presented within, the dosage and duration of treatment of betaine used in Study 3 would yield the best possibility for an increase in nitrite to be noted. If significantly elevated, we may have then had rationale to measure nitrite in samples obtained in Studies 1 and 2. However, this was not the case.

### Physical Activity and Dietary Intake

Subjects were asked to refrain from strenuous physical activity during the 24 hours before test days. Subjects were asked to record all food and drink consumed during the day prior to each test day. Upon receipt of the first diet record, subjects received a copy and were asked to duplicate this intake during the day immediately prior to the subsequent test day. All records were analyzed for total kilocalories, protein, carbohydrate, fat, vitamin C, and vitamin E (Food Processor SQL, version 9.9, ESHA Research, Salem, OR).

### Statistical Analysis

For Study 1, data were analyzed using a 2 (dosage) × 5 (time) analysis of variance (ANOVA). For Study 2, data were analyzed using a 2 (condition) × 2 (pre/post intervention) ANOVA. For Study 3, data were analyzed using one way ANOVA with time as the factor of interest. Data for all studies are presented as mean ± standard error of the mean. Subject descriptive characteristics are presented as mean ± standard deviation. All analyses were performed using JMP statistical software (version 4.0.3, SAS Institute, Cary, NC). Statistical significance was set at P ≤ 0.05.

## Results

Subject descriptive characteristics are presented in Table 1. Dietary data are presented in Table 2, Table 3, and Table 4. No statistically significant differences were noted in any dietary variable in any of the studies (p > 0.05). Results for nitrate/nitrite are presented in Table 5 (Study 1), Table 6 (Study 2), and Table 7 (Study 3). In Study 1, no statistically significant interaction (p = 0.99), dosage (p = 0.69), or time (p = 0.91) effects were noted. In Study 2, no statistically significant interaction (p = 0.57), condition (p = 0.98), or pre/post intervention (p = 0.17) effects were noted. In Study 3, no statistically significant differences were noted in nitrate/nitrite (p = 0.97) or nitrite (p = 0.97) between collection times.

**Table 2:** Dietary data for subjects in Study 1 during the day prior to each test day

Variable	Betaine 1.25 g	Betaine 5.00 g
Kilocalories	2079 ± 295	1812 ± 491
Protein (g)	73 ± 6	71 ± 11
Carbohydrate (g)	277 ± 46	256 ± 71
Fat (g)	79 ± 11	61 ± 19
Vitamin C (mg)	101 ± 28	86 ± 73
Vitamin E (mg)	13 ± 11	15 ± 12

Data are mean ± SEM.

No statistically significant differences noted in any dietary variable (p > 0.05).

Study involved a cross-over design with subjects consuming either 1.25 or 5.00 grams of betaine in a single ingestion.

**Table 3:** Dietary data for subjects in Study 2 during the day prior to each test day

Variable	Pre Placebo	Post Placebo	Pre Betaine	Post Betaine
Kilocalories	1931 ± 183	2147 ± 265	2242 ± 288	2551 ± 325
Protein (g)	115 ± 16	122 ± 16	125 ± 24	138 ± 22
Carbohydrate (g)	249 ± 24	267 ± 41	280 ± 41	320 ± 52
Fat (g)	58 ± 8	69 ± 12	73 ± 12	83 ± 11
Vitamin C (mg)	58 ± 18	76 ± 26	102 ± 34	80 ± 16
Vitamin E (mg)	5 ± 2	4 ± 1	3 ± 1	4 ± 2

Data are mean ± SEM.

No statistically significant condition × pre/post intervention interaction, pre/post intervention, or condition main effects noted for kilocalories (p = 0.69; p = 0.46; p = 0.13), protein (p = 0.94; p = 0.61; p = 0.57), carbohydrate (p = 0.56; p = 0.67; p = 0.17), fat (p = 0.90; p = 0.41; p = 0.14), vitamin C (p = 0.43; p = 0.92; p = 0.33), or vitamin E (p = 0.41; p = 0.86; p = 0.82), respectively.

Study involved a cross-over design with subjects consuming 2.5 grams of betaine or a placebo daily for 14 days; 21 day washout period between each condition.

**Table 4:** Dietary data for subjects in Study 3 during the day prior to each test day

Variable	Pre	Post
Kilocalories	2264 ± 196	2043 ± 236
Protein (g)	146 ± 19	140 ± 20
Carbohydrate (g)	248 ± 42	249 ± 52
Fat (g)	82 ± 8	61 ± 6
Vitamin C (mg)	89 ± 30	82 ± 24
Vitamin E (mg)	7 ± 2	6 ± 2

Data are mean ± SEM.

No statistically significant differences noted in any dietary variable (p > 0.05).

Study involved subjects consuming 6 grams of betaine daily for 7 days.

**Table 5:** Plasma nitrate/nitrite (μmol∙L^-1^) for subjects in Study 1

Dosage	Pre	30 min	60 min	90 min	120 min
1.25 g	34.6 ± 6.9	32.1 ± 7.2	31.8 ± 5.7	28.2 ± 4.6	27.9 ± 5.0
5.00 g	32.9 ± 8.4	29.1 ± 6.9	28.4 ± 8.0	27.3 ± 8.0	28.2 ± 7.4

Data are mean ± SEM.

No statistically significant interaction (p = 0.99), dosage (p = 0.69), or time (p = 0.91) effects noted.

Study involved a cross-over design with subjects consuming either 1.25 or 5.00 grams of betaine in a single ingestion; blood samples collected Pre, 30, 60, 90, and 120 min post intake.

**Table 6:** Plasma nitrate/nitrite (μmol∙L^-1^) for subjects in Study 2

Condition	Pre Intervention	Post Intervention
Placebo	24.3 ± 4.8	17.5 ± 2.4
Betaine	22.4 ± 3.4	19.6 ± 3.1

Data are mean ± SEM.

No statistically significant interaction (p = 0.57), condition (p = 0.98), or pre/post intervention (p = 0.17) effects noted.

Study involved a cross-over design with subjects consuming 2.5 grams of betaine or a placebo daily for 14 days; 21 day washout period between each condition; blood samples collected before (Pre Intervention) and after (Post Intervention) each 14 day period.

**Table 7:** Plasma nitrate/nitrite (μmol∙L^-1^) and nitrite (nmol∙L^-1^) for subjects in Study 3

	Pre Intervention	Post Intervention	30 min post intake	60 min post intake
Nitrate/Nitrite	18.6 ± 3.1	18.2 ± 2.9	18.0 ± 3.2	16.4 ± 3.0
Nitrite	1418.3 ± 137.5	1466.3 ± 146.9	1366.4 ± 148.1	1369.8 ± 200.6

Data are mean ± SEM.

No statistically significant effect noted for nitrate/nitrite (p = 0.97) or nitrite (p = 0.97).

Study involved subjects consuming 6 grams of betaine daily for 7 days; blood samples collected before (Pre Intervention) and after (Post Intervention) the 7 day period; Post intervention, subjects consumed 6 grams of betaine and blood samples were collected 30 and 60 min post intake.

## Discussion

When collectively considering data obtained from the three separate studies, we report that acute or chronic ingestion of betaine does not impact plasma nitrate/nitrite in exercise-trained men. These findings contradict those of Iqbal and coworkers [17,18], and suggest that other mechanisms aside from increasing circulating nitric oxide are likely responsible for the reported ergogenic benefit of betaine supplementation that has been reported by others [5,6]. Of course, our omission of exercise performance measures within the present manuscript may be considered a limitation of this work.

When considering the findings presented here along with those of Iqbal and colleagues [17,18], it is possible that differences in the subject sample may be responsible for the differing results. Specifically, our subjects were young, healthy, exercise-trained men, while those in the Iqbal work were simply reported to be "healthy volunteers". Further work is needed to replicate the findings of Iqbal and colleagues [17,18] in middle and older age adults, to determine if individuals other than healthy, exercise-trained men benefit from betaine supplementation in terms of elevating circulation nitrate/nitrite.

In relation to the above, it has been noted that older individuals may exhibit diminished ability to generate nitric oxide [20,21], which ultimately may result in impaired blood flow [20]. Such findings may have implications in relation to betaine supplementation across different populations. That is, perhaps older individuals with lower basal nitrate/nitrite levels may respond more favorably to betaine supplementation as compared to young and healthy subjects. To our knowledge, no study has yet determined this. However, at least one study has compared plasma betaine levels between younger and older subjects, noting higher levels for older compared to younger subjects [22]. It is presently unknown what the physiological relevance of this difference is in terms of how an individual might respond to betaine supplementation for purposes of increasing circulating nitrate/nitrite. Of course, betaine supplementation may provide health benefits in areas outside of plasma nitrate/nitrite (e.g, reducing homocysteine, reducing the risk of cardiovascular disease and metabolic syndrome) [1], which may warrant its use by a wide variety of individuals--both older and younger. More work is needed to determine the potential health related benefits of betaine supplementation in human subjects.

Dietary supplements that are purported to increase circulating nitric oxide have received a great deal of attention in recent years [16]. The effect that appears to be of greatest interest is that of increasing blood flow to exercising skeletal muscle, as well as regulating muscle tissue atrophy and hypertrophy. Advertisements supporting most such products suggest that an increase in blood flow will result in increased oxygen and nutrient delivery (e.g., amino acids, fatty acids, glucose) to skeletal muscle during exercise. This would then enhance exercise performance, while the increased blood flow will be retained during the post-exercise period, allowing for enhanced exercise recovery--which would ultimately result in muscle hypertrophy. While these hypotheses are interesting, there exists no evidence that such events take place, at least as applied to human subjects consuming oral dietary supplements purported to increase nitric oxide. Even for dietary ingredients reported to result in measurable increases in plasma nitrate/nitrite, such as glycine propionyl-L-carnitine [23,24], additional studies which include functional, rather than just biochemical outcomes, are needed. Without such studies, there is no way of knowing what, if any, physiological effect an increase in circulating nitrate/nitrite has within an *in vivo *system.

Aside from nutritional supplements, other lifestyle methods are available for increasing nitric oxide, including regular exercise training [25,26] and modification of dietary intake to include more fruits, vegetables, and whole grains [Bloomer RJ, Kabir MM, Trepanowski JF, Canale RE, Farney TM: A 21 day Daniel Fast improves selected biomarkers of antioxidant status and oxidative stress in men and women. *Nutrition and Metabolism*. In Press]. Considering the multiple health benefits associated with these activities, if elevating circulating nitric oxide is a goal, it may be best to simply focus on these activities.

## Conclusion

Acute or chronic ingestion of betaine by healthy, exercise-trained men does not impact plasma nitrate/nitrite. It is possible that betaine supplementation by older and/or deconditioned individuals, or possibly by women, may result in elevated nitrate/nitrite levels in plasma. Additional work is needed to confirm such a hypothesis. Based on our findings, in regards to the recently reported ergogenic properties of betaine [5,6], mechanisms aside from an elevation in nitrate/nitrite are likely responsible for these effects.

## Competing interests

RJB has received research funding or acted as consultant to nutraceutical and dietary supplement companies. All other authors declare no competing interests.

## Authors' contributions

RJB was responsible for the study designs, overseeing data collection, biochemical work, statistical analysis, and preparation of the manuscript. TMF, JFT, CGM, and REC were responsible for data collection/entry and assistance with manuscript preparation. All authors read and approved the final manuscript.
